# Visual analysis of the research frontiers, hotspots and trends of exercise therapy intervention in tumor-related sleep-wake disorders

**DOI:** 10.3389/fonc.2024.1392844

**Published:** 2024-04-29

**Authors:** Jilei Han, Jiachen Zhang, Litao Zhang, Juan Guo, Xitao Peng, Chenlin Ying, Zhiqing Li, Mu Li, Lihua Chang, Yani Zhang

**Affiliations:** ^1^ Sports Center, Xi'an Jiaotong University, Xi’an, China; ^2^ Xi'an Jiaotong University Health Science Center, Xi’an, China; ^3^ Department of Dermatologic Allergies (Allergies), Tianjin Academy of Traditional Chinese Medicine Affiliated Hospital, Tianjin, China; ^4^ Department of Physical Education, The High School Affiliated to Xi’an Jiaotong University, Xi’an, China; ^5^ Teaching Center for Physical Education, Xi'an Jiaotong University City College, Xi’an, China; ^6^ Department of Obstetrics and Gynecology, the Second Affiliated Hospital of Xi'an Jiaotong University, Xi’an, China; ^7^ Department of Preventive Health and Community Service, the Second Affiliated Hospital of Xi'an Jiaotong University, Xi’an, China; ^8^ Library of the Academic Affairs Department, the Second Affiliated Hospital of Xi'an Jiaotong University, Xi’an, China

**Keywords:** sleep-wake disorders, visualization, tumor-related, exercise therapy, bibliometric analysis platform.

## Abstract

**Objective:**

To systematically understand the research frontiers, hotspots and development trends of exercise therapy in the intervention of tumor-related sleep-wake disorders, and to provide scientific basis for follow-up research.

**Methods:**

Downloaded the original research papers on February 26, 2024, from the Web of Science core collection database, on tumor-associated sleep-wake disorders. The data that met the inclusion criteria were imported into the Bibliometric Analysis Platform (http://biblimetric.com), CiteSpace 6.3.R1 and VOSviwer1.6.20 software for visual analysis, and imported into Excel2021. Scientometric analysis was performed with Oringin2021 and PyCharm Community Edition 2022.1.3.

**Results:**

A total of 512 original research papers on tumor-related sleep-wake disorders were obtained. The most influential countries in the subject area are the United States, Spain and German, the institutions are the University of California System, Sun Yat Sen University and Northwestern University, et al., the authors are Berger AM, Aaronson NK, Bower JE, et al., and the journals are *Cancer*, *Brit J Cancer* and *Cancer Nurs*. The co-cited references suggest that the current research frontier in the field mainly involves the level, place and program of exercise therapy, including the relationship between physical activity, sedentary behavior and cancer prevention and control. The results of co-occurrence keyword network analysis showed that quality of life, physical activity, breast cancer, exercise, fatigue, and survivors may be the research hotspots in this field, with breast cancer, health, aerobic exercise, adults, and chemotherapy being the most popular.

**Conclusions:**

The number of papers published and the research enthusiasm in this field show a steady upward trend. However, there is a lack of influential institutions and scholars, and there is relatively little research collaboration across countries/regions/institutions. The scientific research influence of institutions and scholars in most European and American countries/regions is significantly ahead of that of institutions and scholars in Asian and African countries/regions. But Sun Yat Sen University in China is a relatively active and influential scientific research institution in recent years, which is worthy of attention. In addition, the research frontier of this discipline is the level, place and program of exercise therapy auxiliary intervention, and the research hotspots involve breast cancer, health, aerobic exercise, adults, chemotherapy, et al. Their clinical efficacy needs to be further demonstrated in multi-center, large-sample and high-quality prospective studies.

## 1 Introduction

Sleep-wake disorders are one of the most common comorbidities in cancer patients (1, 2), and there is a bidirectional link between them (3). It typically presents in the form of “clusters” of symptoms, including pain, anxiety, depression, fatigue, and decreased morning energy (4, 5). The severity of this syndrome is positively correlated with cognitive dysfunction, quality of life, cancer survival, and disease burden (6–8) and, if it does not achieve long-term remission, can cause insomnia (9), complicating the management of sleep-wake disorders in oncology (5). The study concluded that: sleep-wake disorders in cancer patients may be independent of the time of diagnosis of the disease and the location of the tumor, but independently related to their quality of life (10). Sleep-wake disorders are sometimes the most prominent or only initial symptom in patients with autoimmune encephalitis, and this is one of the key factors contributing to the delay in diagnosis (11). Sleep deprivation-induced GABA can promote the proliferation and migration of colon tumors through the miR-223-3p endogenous and exosomal pathways (12), and acute and chronic sleep deprivation can not only affect the occurrence and progression of circulating tumor cells (CTCs) through drugs that modulate sleep-wake circadian rhythms (e.g., regular sleep patterns, bedtime, and wake time) (13). It can also induce immunosuppression in the tumor microenvironment by impairing immune surveillance mechanisms, thereby accelerating disease progression in patients with hepatocellular carcinoma (14). In addition, it is the most serious complication of prostate cancer patients treated with androgen deprivation therapy (15). However, in clinical practice, comorbid sleep-wake disorders in cancer patients are often overlooked by clinicians and patients (16) and are associated with poor prognosis (7, 14, 17, 18).

For patients with tumors and sleep-wake disorders, we need to prioritize improving their sleep quality, followed by educating and training relevant personnel on pharmacological and nonpharmacologic treatments for sleep-wake disorders (19). Previous treatments for these patients have included pharmacological, psychological, behavioral, and motor interventions. Studies have found that complementary therapies such as physical exercise (e.g., aerobic exercise, resistance training, running, et al.), psychological counseling (e.g., cognitive behavioral therapy, psychoeducational interventions, et al.), and psychosomatic interventions (e.g., yoga, mindfulness, hypnosis, et al.) can significantly improve patients’ sleep and quality of life (20). Omega-3, cannabidiol, acupuncture, and cognitive behavioral therapy may help improve sleep quality (21–23). Exercise therapy combined with mindfulness-based stress reduction is effective in reducing the incidence of sleep-wake disorders in cancer patients (19). Flexible living programs can significantly improve the quality of life of patients with brain tumors and their family caregivers (24). Interestingly: fatigue, morning and evening energy levels, and severity of sleep-wake disturbances have also been found to be significantly correlated with daily physical activity levels and quality of life in cancer patients (25, 26). They believe that increasing the level of daily activities in individuals, even low-intensity daily household activities, repetitive sitting, walking, et al., can also safely and effectively improve sleep-wake disorders in different populations (27–29), and noted that chemotherapy combined with exercise therapy can effectively reduce adverse effects such as chemotherapy-induced peripheral neuropathy and sleep disturbance (29). However, the relationship between sleep-wake disorders and specific tumor diseases is still unclear, and the optimal dosage of therapeutic drugs, the clinical efficacy of combination drugs (21, 22), and the optimal strategy for implementing precision medicine (13),et al, still need to be further explored or demonstrated. There is still no conclusion on the adjuvant intervention mode and level of exercise therapy, the mechanism of action, the clinical application field, and the doctor’s cognition. Therefore, it is of great significance to conduct in-depth investigation studies and clinical trials of tumor-related sleep-wake disorders, and to explore the evaluation, management, and treatment of new technologies.

Bibliometrics is a discipline that takes the bibliometric system and bibliometric characteristics as the research object, and uses econometric research methods such as mathematics and statistics to explore the distribution structure, quantitative relationship, change law and quantitative management of bibliometric information, and then explores some structures, characteristics, and laws of science and technology (30). Visual analysis is to use a multivariate, time-sharing, and dynamic citation analysis visual language to visualize the vast literature data of a knowledge field, and through an ingenious spatial layout, the evolution process of the field is concentrated on a knowledge graph of the citation network, and the research frontiers represented by citation node literature and co-citation clustering as the knowledge base on the graph are automatically identified, reflecting the interpretability of the graph itself (31).

At present, the number of papers related to the adjuvant intervention of exercise therapy in the field of tumor-related sleep-wake disorders is gradually increasing, which reflects the increasing attention of scholars in this field. However, no scholars have conducted bibliometric studies and visual analyses on relevant studies in this field. In order to understand the application status and development trend of digital technology in this field, and to explore the research frontiers and hotspots of digital technology in this field, this study intends to conduct bibliometric research and visual analysis of the current status of original research literature related to this topic in the core collection database of Web of Sciences, aiming to explore its research frontiers, hotspots and development trends, provide reference data for follow-up research, and provide new ideas and new methods for relevant departments to formulate sports therapy auxiliary intervention programs.

## 2 Data and methodology

### 2.1 Data collection

**Figure 1:**
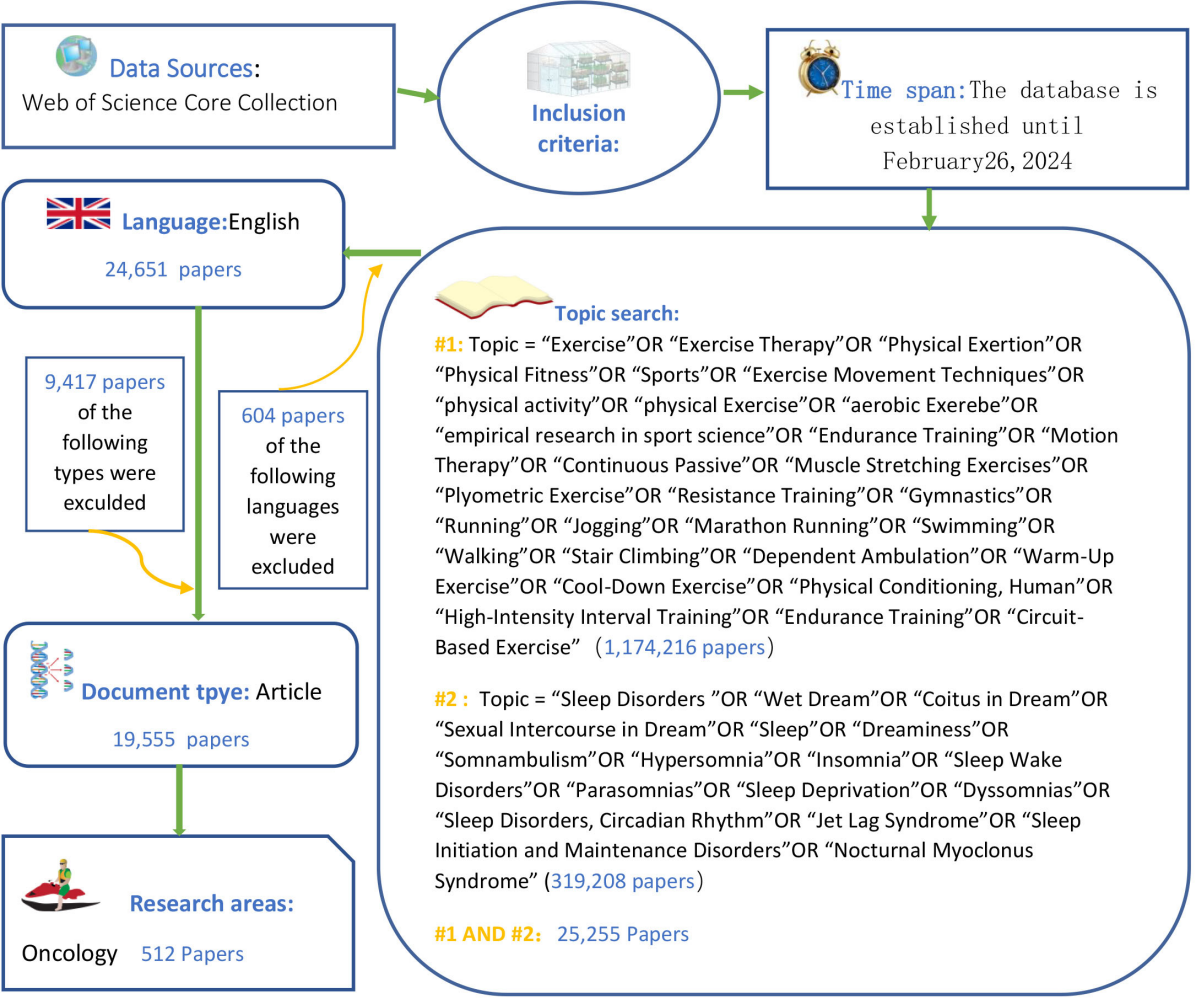
Literature Search Strategy.

Log in to the Web of Science database platform (http://apps.webofKnowledge.com/), select the core collection database, and refer to the existing literature and the procedures and standards of scientometric analysis to formulate a literature retrieval strategy (31–34), as shown in **Figure 1**.

### 2.2 Research method

Entered the count items, which meet the inclusion criteria, into Excel2021, such as publication time, citation frequency, and number of articles published by different subjects (e.g., authors, institutions, countries/region, et al.). Used Oringin 2021 and PyCharm Community Edition 2022.1.3 to map the publication time and citation frequency characteristics of publications, along with the development trend map in the next 5 years. The H-index obtained from the WoSCC webpage is used to assess the scientific impact of academic outputs of different subjects (35).Then import the publication information downloaded from the website into CiteSpace6.3R1, VOSviwer1.6.20 or the Bibliometric Analysis Platform (http://biblimetric.com). To understand the development status of the discipline by drawing the scientific research co-authorship map of countries/regions, institutions and authors, to explore the research hotspots and development trends of the subject by drawing the co-occurrence keyword map and the timeline diagram of co-occurrence keyword clustering, to analyze the high-impact journals in the subject area by drawing the co-cited journal map, and to understand the research frontier and its evolution path of the subject area by drawing the co-cited reference cluster map. We draw the scientific map, and the specific parameters are shown in the parameter information in the upper left corner of the picture.

### 2.3 Interpretation methods of visual knowledge graph

In the scientific knowledge graph, each node represents an element, representing the country/region, institution, author, co-cited author, co-cited journal, co-cited reference, and co-occurrence keyword. There is a positive correlation between node size and the number of published papers, co-occurrence frequency or co-citation frequency. The node color represents the time when it first appears, and the evolution process from cool color to warm color represents the development path of its appearance time from far to near. The outermost purple circle of a node represents the betweeness centrality≥0.1, which is generally considered to be a key node in its domain, that is, it has an important “bridge” role. The annual rings of a node represent its citation history, the size of the annual rings reflects the number of times it has been cited, the color of the annual rings represents the citation time, and the thickness of the annual rings is proportional to the number of citations in the corresponding time partition. The diameter of the connection between nodes is positively correlated with the number of co-authored papers, co-occurrence frequency or co-citation frequency. The color of the connection represents the time when it was first co-authored, co-appeared, or co-cited, and the evolution process of the network connection from cool to warm represents the evolution of the research field from far to near in time (31, 32).

In a clustered view, the larger the number of members contained in a clustered cluster, the smaller its number. (1) The larger the Modularity value, the better the clustering effect, and when Q>0.3, the structure of each clustered cluster is significant. (2) The larger the Silhouette value, the higher the similarity of the members within the clustering cluster (when there are few members within the cluster, its reliability will decrease); when the Silhouette > 0.7, it indicates that the reliability of the clustering results is higher, when it is 0.5~0.7, the clustering results are considered reasonable, and the closer the Silhouette value is to 1, the higher the homogeneity within the cluster. (3) The more nodes contained in the clustered cluster, the more important the research field involved in the clustered cluster, and its time span is positively correlated with the duration of its research popularity. (4) The more burst nodes contained in the clustered cluster, the more active the research content involved in this field is, and it also suggests that it may be an emerging trend in the field of research (33, 34).

In the emergent map, the burst node is filled with red in the corresponding burst year, and its time span is positively correlated with the duration of the research enthusiasm of the node. In the timeline view, nodes in the same clustered cluster are placed on the same horizontal line, and the node occurrence time is placed at the top of the graph, and the nodes on the right are more recent. It is worth noting that burst detection is often used to find nodes with “bridge” role in different clustered clusters in cluster maps and time views (36).

## 3 Results

### 3.1 Search results

**Figure 2:**
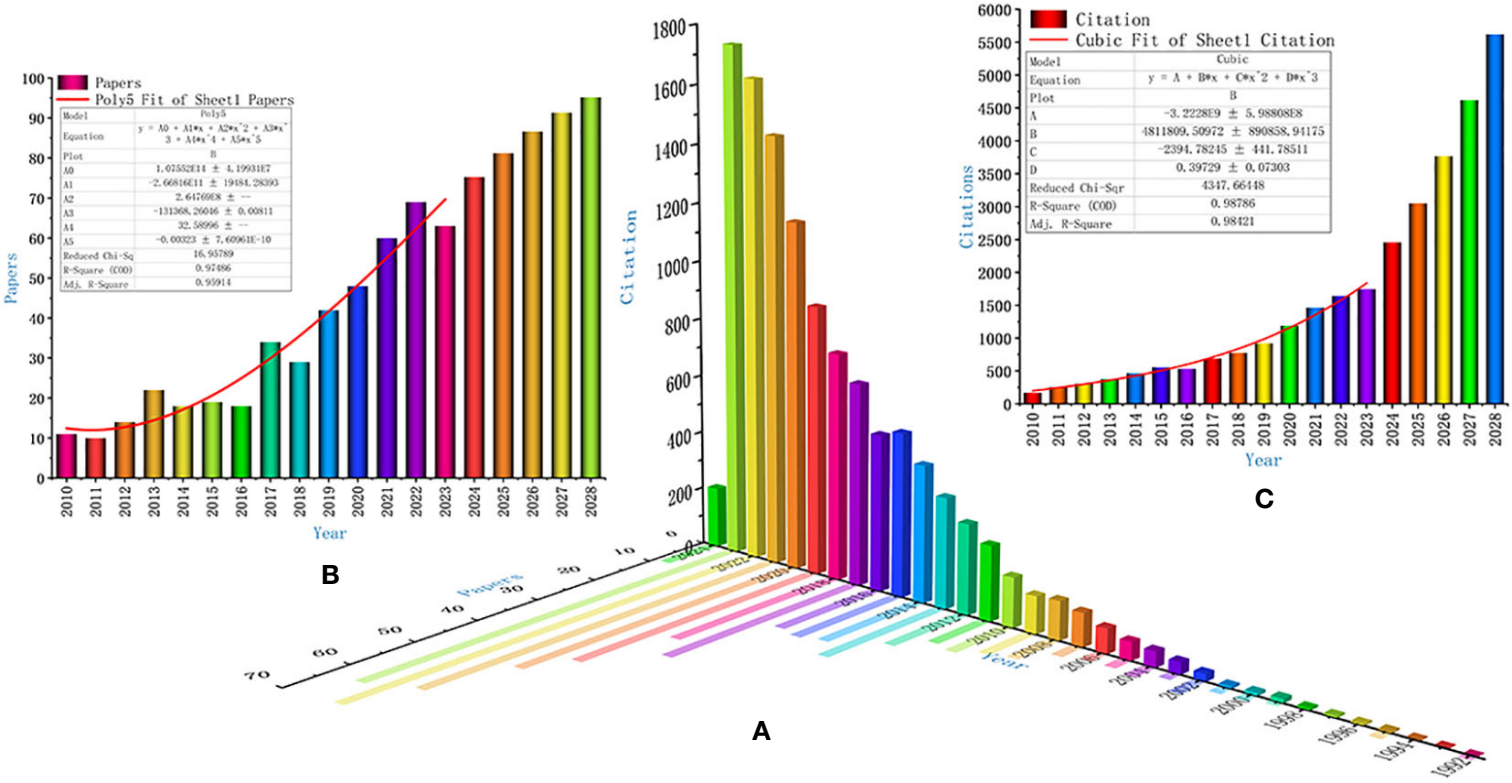
Publication Time and Citation Distribution Characteristics of Publications **(A)**, Publication **(B)** and Citation Frequency **(C)** in the Next 5 Years.

We found a total of 512 relevant publications, which were cited a total of 12013 times, with an h-index of 57. The distribution characteristics of their publication time and citation times are shown in **Figure 2A**, and predict their development trends over the next 5 years as shown in **Figures 2B, C**.

### 3.2 Network analysis of co-cited references

**Figure 3:**
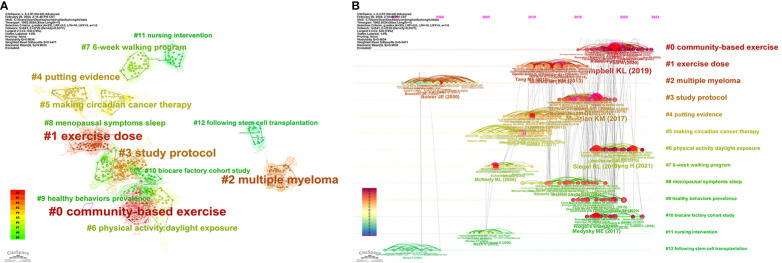
Co-cited reference cluster analysis map **(A)** and timeline view **(B)** drawn by Citespace.

There are a total of 17091 co-cited references, of which only 27 are cited more than or equal to 28 times. The clustering map of the co-cited references was well structured, and the internal consistency of each cluster was high (Q=0.8634, S=0.9471, **Figure 3A**). The 14 clusters the evolution process of cluster names from cool to warm colors represents the evolution path of the research frontier in this discipline from far to near in time) were constructed into six main research trends(**Figure 3B**). The 1^st^ item: identified in 1997 and mainly involved “following stem cell transplantation” (#12). The 2^nd^ item: identified in 2015 as a reference to “life” (#26). It continues to be the most active research area throughout the development of the subject area. The 3^rd^ item: identified separately in 2012- 2019. It mainly involves the intervention research on cancer fatigue and sleep quality in cancer patients, including exercise level, venue, program, et al. (#0, #1, #3, #6, #8, #9), and is also the most popular and cutting-edge research content in this field. The 4^th^ item: identified in 2008-2010. It primarily deals with the clinical practice of exercise therapy (#4, #5). The 5^th^ item: identified separately in 2005-2007. It primarily involves studies of nursing intervention programs in exercise therapy (#7, #11). The 6^th^ item: Identified in 1999, mainly related to “multiple myeloma”. Moreover, the emergent map of co-cited reference clustering suggests (**Supplementary Figure 1**): Campbell et al. (37) published in *Med Sci Sports Exerc* in 2019 had the highest number of co-citations (research popularity), and Mustian et al. (38) published in *JAMA Oncol* in 2017 was the most cited and pointed out that the combination of exercise intervention and psychotherapy can significantly reduce chronic renal failure during and after cancer treatment, and recommended that clinicians should use it as the first choice for first-line treatment of cancer. In addition, the current research frontiers in this field also focus on the relationship between physical activity, sedentary behavior, and cancer prevention and control (39), as well as the active assessment and management of the medical and psychosocial needs of cancer patients at all stages (40).

### 3.3 Co-occurrence keyword network analysis

**Figure 4:**
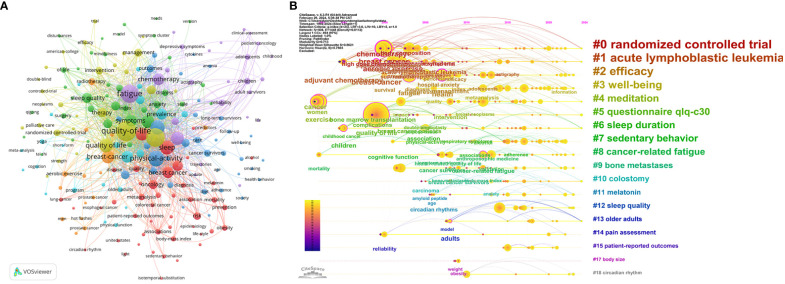
Scientific knowledge graph of co-occurring keywords drawn by VOSviewer **(A)** and timeline diagram of keywords **(B)** drawn by Citespace.

The high-frequency co-occurrence keywords of the subject area reflect its main research hotspots. A total of 508 keywords were extracted by CiteSpace software (**Figure 4A**), of which 208 keywords appeared at least 5 times, and the top 10 keywords in terms of frequency were: quality of life (253 times), physical activity (158 times), and breast cancer (120 times), exercise (112 times), fatigue (92 times), survivors (75 times), women (62 times), depression (58 times), Prevalence (57 times), health (56 times), sleep (56times). Therefore, we believe that these co-occurrence keywords may be the research hotspots in the field of tumor-related sleep-wake disorders. In addition, **Figure 4A** also suggests that the top 10 co-occurrence keywords of subject influence are: breast cancer (betweeness centrality: 0.21), health(0.21), aerobic exercise (0.2), adults (0.17), chemotherapy (0.16), cancer(0.15), quality of life (0.13), exercise (0.12), fatigue (0.11), cancer-related fatigue (0.11), children (0.11). In the timeline diagram of co-occurrence keyword clustering (**Figure 4B**), the transformation process of cluster names from cool to warm colors reflects the development trend of research hotspots in this discipline. The co-occurrence keyword emergence map suggests that “adjuvant chemotherapy” has been the long-term concern of scholars (1995-2012), and “fatigue” has been the most popular (2012-2015). However, at present, there is no co-occurrence keyword with the highest research enthusiasm and active state.

### 3.4 Scientific Co-authorship Network Analysis

#### 3.4.1 Countries/regions

**Figure 5:**
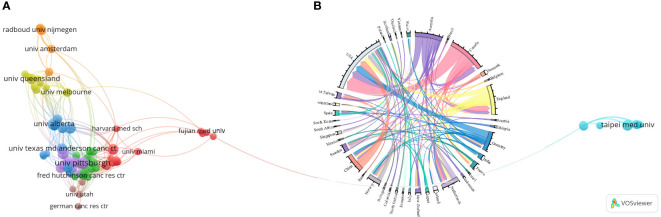
Research co-authorship map of countries/regions **(A)** and institutions **(B)**.

Among the 47 countries/regions which published papers, only 18 countries/regions have published 5 papers or more, among which the United States(251 papers), China(74 papers), and Canada(55 papers) have published the most papers. In addition, the countries with academic influence (betweeness centrality) greater than 0.1 were USA (0.84), Spain (0.17), England (0.17), and Germany(0.15), refer to **Figure 5A**. However, China, along with countries such as England, Australia and Netherlands, is currently the most active cluster.

#### 3.4.2 Institutions

Among the 1124 institutions that published papers, only 72 institutions published 5 papers or more (**Figure 5B**). The University of California System was once the most influential institution in its field, ranking first in the world in terms of the number of published papers (32 papers), the number of co-authored papers (28 papers), and its betweeness centrality (0.2) in the institutional research co-authorship network. The second and third largest institutions in the world in terms of the number of papers published are the University of Texas System (23 papers), Harvard University (22 papers), and Pennsylvania Commonwealth System of Higher Education Pcshe (PCSHE, 22 papers). In addition, Sun Yat Sen University (0.17) and Northwestern University (0.16) ranked second and third respectively in terms of influence in the institutional co-authorship network. However, the number of papers co-authored and published by the two institutions is only 7 and 5. The University of Amsterdam, which used to be the institution with the highest burst intensity, is currently the university of Pittsburgh with the highest burst intensity, along with the German Cancer Research Center (DKFZ), Harvard University, Fred Hutchinson Cancer Center, University of The cluster of 12 institutions, including Washington, remains the most active research institution.

#### 3.4.3 Authors

Among the 3209 authors who published papers, only 20 authors published 5 papers or more. Courneya KS, Lin CC, Miaskowski C, et al. published the largest number of papers, and Courneya KS, Miaskowski C and Levine JD published the highest number of co-authored papers. Bower JE, Buysse DJ, and Berger AM had the highest co-citation frequency, and Berger AM, Aaronson NK, and Bower JE had the highest co-citation impact. The author’s co-authorship spectrum emergence-analysis found that Miaskowski C was the only and still active author in the subject area. The authors co-cited map emergent analysis (**Supplementary Figure 2**) found that: Mock V had the highest burst intensity (10.29) and that it had been highly watched for the longest time (1999-2014) along with Dimeo F, Broeckel JA, and Morrow GR, et al.; Campbell KL, Jemal A, Harris PA, Abrahams HJG, Sung H, Miller KD, and Irwin MR continue to be the authors who are currently receiving a high level of interest.

Courneya KS, Miaskowski C, and Levine JD co-authored the highest number of papers. Bower JE, Buysse DJ, and Berger AM had the highest co-citation frequency, and Berger AM, Aaronson NK, and Bower JE had the highest co-citation impact. The author’s co-authorship spectrum emergence-analysis found that Miaskowski C was the only and still active author in the subject area. The authors were found to have the highest burst intensity (10.29) with Mock V, which together with Dimeo F, Broeckel JA, and Morrow GR and others were the most highly visible (1999-2014), Campbell KL, Jemal A, Harris PA, Abrahams HJG, Sung H, Miller KD, and Irwin MR continues to be the author of the highest interest at present.

### 3.5 Network analysis of co-cited journals

Among the 97 journals that published papers, only 24 journals published 5 papers or more, with *Support Care Cance* (71 papers), *Cancer Nurs* (42 papers), *Psycho-Oncol* (30 papers) publishing the largest number of papers, *J Clin Oncol* (366 papers), *Support Care Cancer* (320papers), and *Cancer* (301 papers) was the most frequently cited, with *Cancer* (betweeness centrality: 0.1), *Brit J Cancer* (0.09) and *Cancer Nurs* (0.08) having the highest impact. The emergent map of the co-cited journals (**Supplementary Figure 3**) showed that *Cancer* had the highest emergence intensity (10.47) and the longest duration (1992-2013). *Cancers* also had the highest emergence-intensity (9.15), with *Medicine*, *JAMA Netw Open*, *Nutrients*, *Int J Env Res Pub He*, *SCI Rep-UK*, and *Jnci Cancer Spect* remain the most active journals currently (2021-2024).

## 4 Discussion

Our findings show a clear upward trend in both the number of papers published and the total number of citations in this subject area from 2019 to 2023. We also predict that this significant upward trend will continue over the next 5 years, indicating that the subject area is receiving a lot of attention from scholars around the world. In addition, although the United States ranks first in the world in the number of papers published, the number of papers published in this field in China in the past three years and the total number of citations of papers have been more than double the average annual number of papers in the previous period. Importantly, our research also found that Sun Yat-sen University in China is second only to Northwestern University in the United States in terms of global academic power, and it is still active in research. This may be related to the fact that the Chinese government attaches great importance to the prevention and treatment of chronic diseases and malignant tumors caused by the aging of the social population (41, 42).

### 4.1 Research frontiers and evolutionary paths

Through the analysis of the co-cited reference network, our study identified the following research frontiers in this discipline: the efficacy goals of exercise therapy in the adjuvant treatment of patients after tumor resection (43), physical behavior and activity levels (44, 45), exercise levels (45, 46), exercise patterns (46), exercise sites (47), treatment regimens (48–50), and the clinical efficacy and mechanism of exercise therapy in improving sleep therapy and living standards in chemotherapy patients (51). Typical representative studies suggest: cancer patients should be helped to establish healthy eating behaviors and develop individualized exercise behavior and exercise level intervention strategies (44, 45). Although moderate-intensity walking is effective in improving sleep in cancer patients (45), aerobic exercise combined with resistance exercise may be more clinically effective (46), and exercise therapy interventions are best tailored to the patient’s physical impairments at baseline, such as: loss of muscle strength, cardiopulmonary decline, or sleep disturbances, et al (48). In addition, insomnia is a predictor of cancer progression and quality of life, and vestibular stimulation is particularly appropriate for the treatment of its accompanying symptoms (49). Menopausal symptoms are the strongest predictors of menopausal symptoms in patients with gynecologic cancers, and assessment of menopausal symptoms and sleep quality during and after cancer treatment, timely intervention (51), reduction of exercise-induced discomfort, and control of insomnia can effectively improve their health-related quality of life (43). We also found that “life” is the research frontier which runs through the subject area from beginning to end, and it is still active. Recent studies have concluded that the quality of life of cancer patients is positively correlated with their income and physical activity level (52), and the symptom management system (SMILE) can improve their life and have a positive clinical effect on fatigue and sleep disturbance after adjuvant or palliative chemotherapy (53). In short, mastering the above research frontiers in this discipline will help clinicians and relevant health care personnel to formulate targeted exercise therapy intervention programs and measures according to patients’ personality characteristics, so as to scientifically guide and supervise patients to implement exercise therapy scientifically and efficiently, which will be of great significance for managing patient compliance and promoting the healthy development of the discipline.

### 4.2 Research hotspots and development trends

In this study, we identified a total of 111 keywords that appeared 5 times or more. The results showed that the co-occurrence keywords, such as: quality of life, physical activity, breast cancer, exercise, fatigue, and survivors, appeared most frequently. So we thought that thy may have been the research hotspots in this subject area. From the perspective of the connection strength and node size of co-occurrence keywords in the scientific knowledge graph, we also found that breast cancer, quality of life, exercise, physical activity, fatigue, chemotherapy, health, adjuvant chemotherapy, etc. have had significant academic influence in this discipline. Therefore, we believe that they deserve special attention and in-depth study. A representative study suggests that symptom clusters in Asian-American breast cancer survivors may improve with the duration of individual/group/supportive technology interventions (54). High-intensity aerobic interval training (HIIT) combined with resistance training (RES) has been shown to improve adverse effects such as depression, daytime sleepiness, and insomnia in patients undergoing radiotherapy and chemotherapy for rectal cancer (55). Regular endurance training combined with resistance training can improve mental health, sleep, quality of life, and physical health in cancer survivors (56). Providing telehealth cognitive and behavioral training for insomnia may also help improve sleep quality in cancer survivors in rural or economically disadvantaged areas (57). Moderate- to high-intensity physical activity may be of greater benefit to patients with bone metastases. Reducing sedentary behavior may be a key goal for patients with a history of fractures (58). Oncology nurses play a key role in providing education about the benefits of exercise, overcoming barriers to physical activity, and timely referral (59). In addition, it can be seen from the timeline diagram of co-occurrence keyword clustering that the transformation process of cluster names from cold to warm reflects the development trend of research hotspots in this discipline from far to near in time, and also suggests that the research topics related to “radiotherapy-related fatigue”, “controlled trial” and “nutritional state metabolism” are still the current research hotspots. They may be involved in survivors, prevalence, breast cancer, physical activity, sleep, fatigue, health, depression, et al. This is also consistent with the visualization results of the co-occurrence keyword scientific knowledge graph.

### 4.3 Limitations

Although our study elaborates the research status of exercise intervention in tumor-related sleep-wake disorder through bibliometric and visual analysis, and provides a relatively objective and detailed basis, there are still some unavoidable limitations. First of all, the literature data of this study came from the WoSCC database, and there are still some excellent publications that have not been included. Second, our study excluded non-English publications and papers other than original research.

## 5 Conclusion

Overall, in the past five years, the number of publications in research on exercise intervention for tumor-associated sleep-wake disorders has shown a continuous upward trend. The United States is a pioneer country in this field, and China is a relatively active country in the past three years, which has made important contributions to the development of this field. However, we also see that the scientific research collaboration of institutions and individuals in Europe and the United States is also crucial for the productivity of research on exercise therapy intervention in tumor-related sleep-wake disorders, which will also be an important force in future research in this field. In summary, this study provides a scientific basis for follow-up research in the field of exercise therapy intervention in tumor-related sleep-wake disorders through quantitative research and visual analysis of the publication trends, research frontiers, research hotspots and scientific research collaborations, so that readers can quickly and effectively obtain relevant knowledge and important information in the field.

## Data availability statement

The raw data supporting the conclusions of this article will be made available by the authors, without undue reservation.

## Author contributions

JH: Writing – original draft, Conceptualization, Data curation, Methodology, Software, Visualization. JZ: Writing – original draft, Conceptualization, Data curation, Methodology, Software, Visualization. LZ: Writing – review & editing, Resources, Supervision, Validation. JG: Data curation, Resources, Software, Validation, Writing – review & editing. XP: Conceptualization, Funding acquisition, Supervision, Validation, Writing – review & editing. CY: Funding acquisition, Resources, Validation, Writing – review & editing. ZL: Funding acquisition, Supervision, Validation, Writing – review & editing. ML: Data curation, Funding acquisition, Visualization, Writing – review & editing. LC: Funding acquisition, Resources, Supervision, Validation, Writing – review & editing. YZ: Writing – review & editing, Conceptualization, Data curation, Methodology, Software, Supervision, Visualization.

## Supplementary material

Supplementary Figure 1 Emergent analysis of the clustering map of co-cited references
