# Supplementary figures and images for: Visual analysis of the research frontiers, hotspots and trends of exercise therapy intervention in tumor-related sleep-wake disorders
Source: Front Oncol. 2024 Apr 29;14:1392844. doi: 10.3389/fonc.2024.1392844 (PMC11089126; doi:10.3389/fonc.2024.1392844)

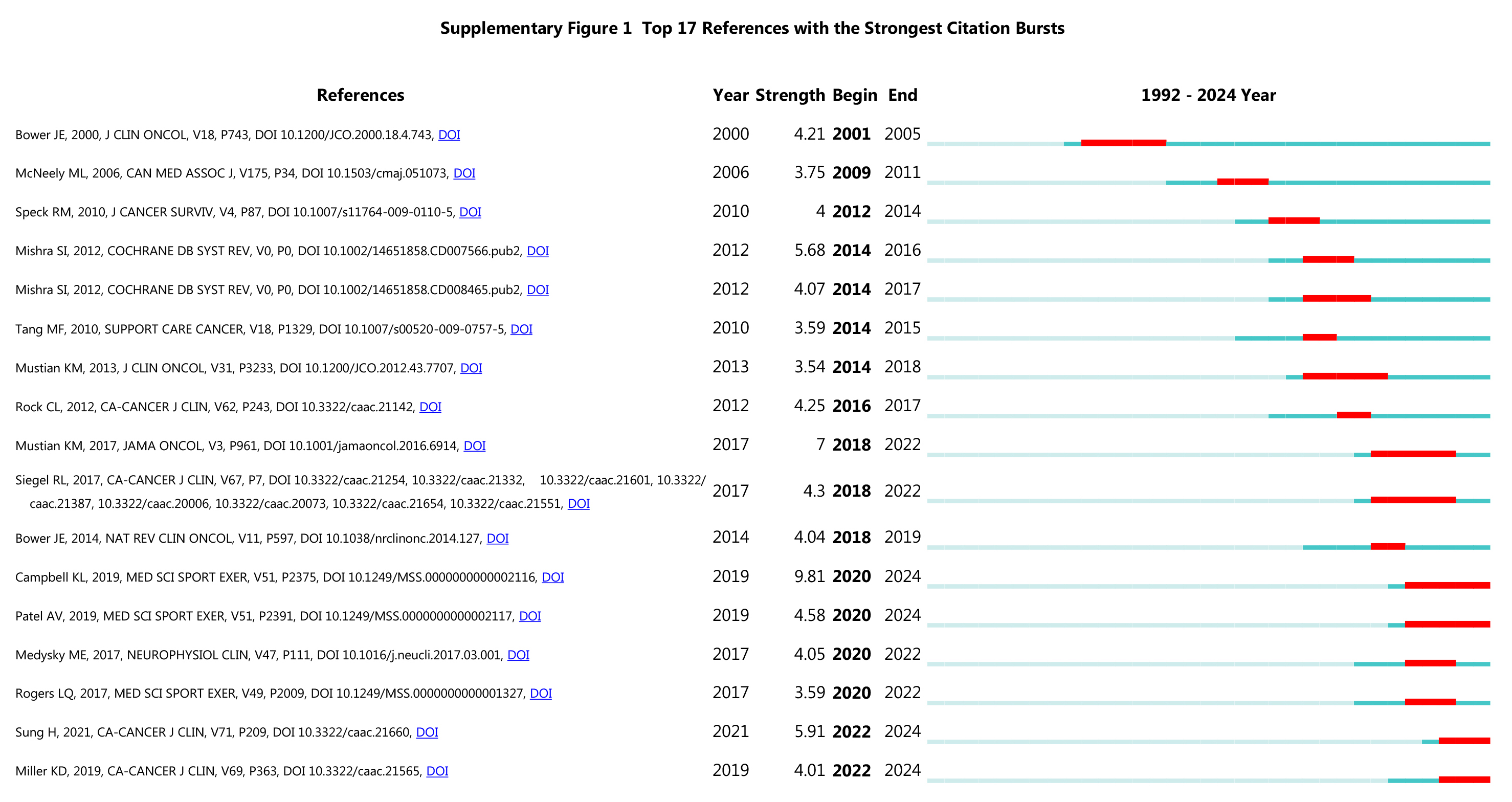

Supplement: Supplementary Figure 1 — Emergent analysis of the clustering map of co-cited references [file Image_1.jpeg]

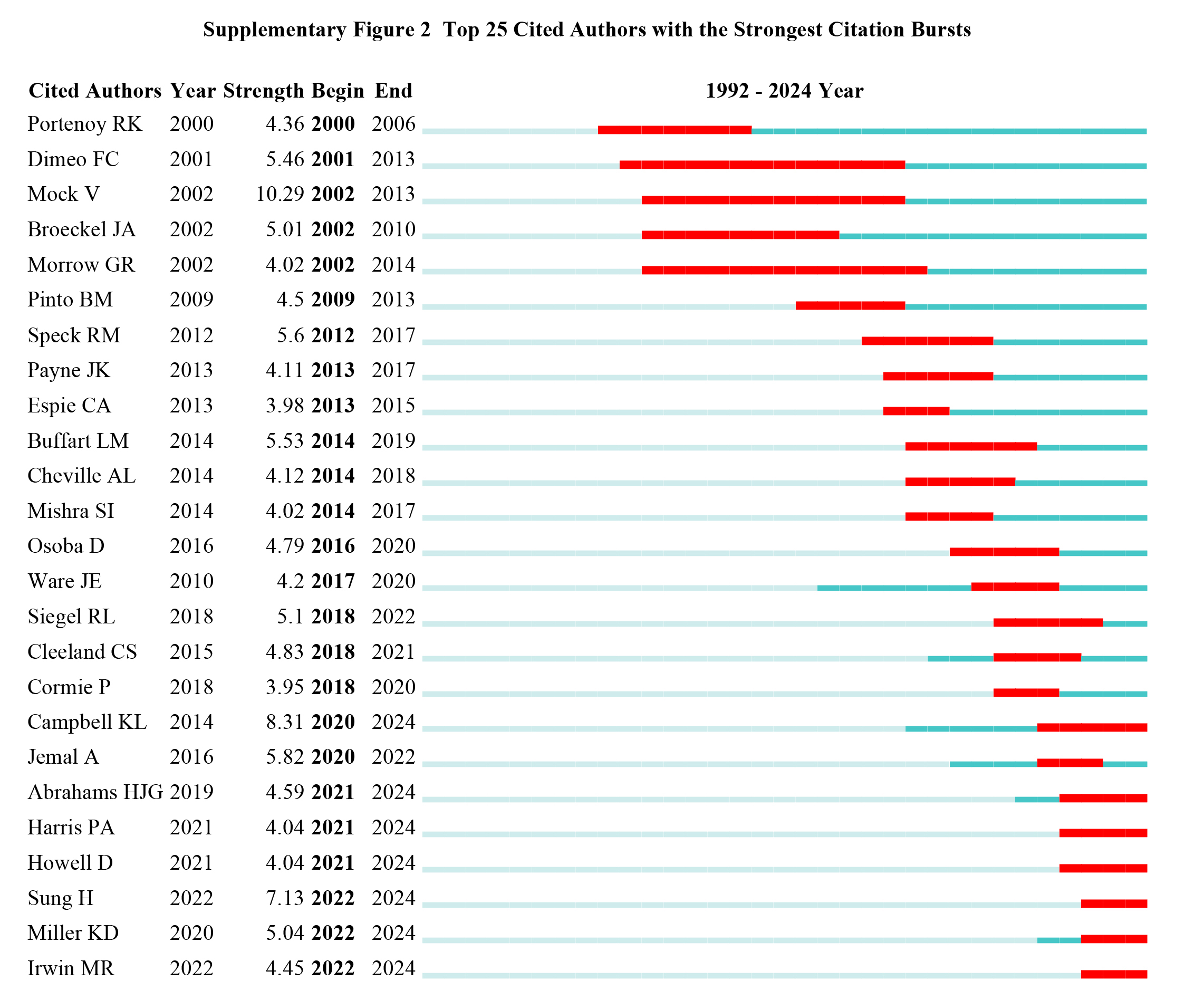

Supplement: Supplementary file 2 [file Image_2.jpeg]

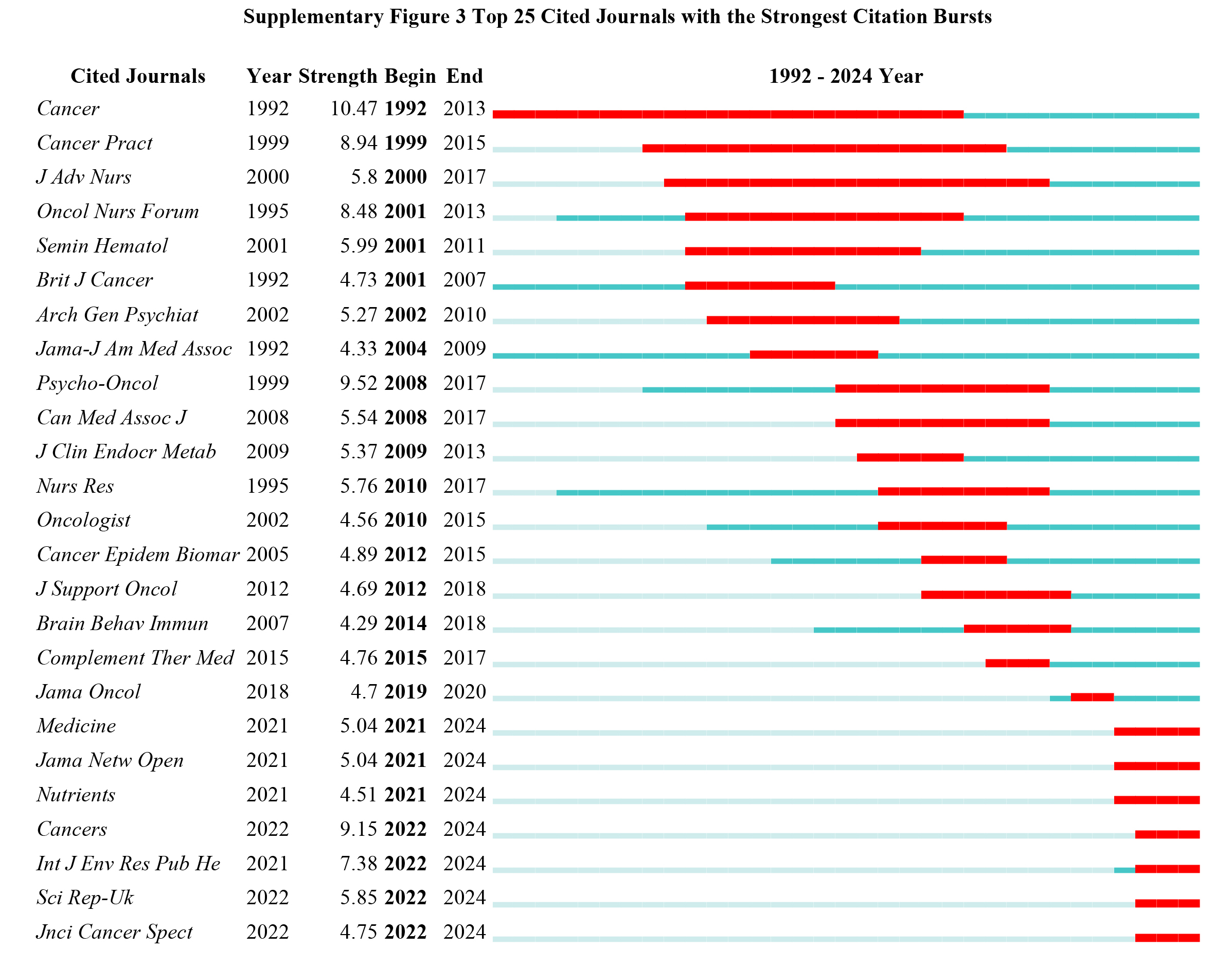

Supplement: Supplementary file 3 [file Image_3.jpeg]
